# Recent Advances by In Silico and In Vitro Studies of Amyloid-β 1-42 Fibril Depicted a S-Shape Conformation

**DOI:** 10.3390/ijms19082415

**Published:** 2018-08-16

**Authors:** Daniel Miguel Ángel Villalobos Acosta, Brenda Chimal Vega, José Correa Basurto, Leticia Guadalupe Fragoso Morales, Martha Cecilia Rosales Hernández

**Affiliations:** 1 Laboratorio de Biofísica y Biocatálisis, Sección de Estudios de Posgrado e Investigación, Escuela Superior de Medicina, Instituto Politécnico Nacional, Plan de San Luis y Díaz Mirón s/n, Casco de Santo Tomás, Distrito Federal 11340, Mexico; daniel2015mip@gmail.com (D.M.Á.V.A.); brendachimal27@hotmail.com (B.C.V.); lety.23fm@gmail.com (L.G.F.M.); 2 Laboratorio de Modelado Molecular y Bioinformática, Sección de Estudios de Posgrado e Investigación, Escuela Superior de Medicina, Instituto Politécnico Nacional, Plan de San Luis y Díaz Mirón s/n, Casco de Santo Tomás, Distrito Federal 11340, Mexico; corrjose@gmail.com

**Keywords:** Alzheimer’s, beta amyloid 1-42, S-shape, oligomerization

## Abstract

The amyloid-β 1-42 (Aβ1-42) peptide is produced by proteolytic cleavage of the amyloid precursor protein (APP) by sequential reactions that are catalyzed by γ and β secretases. Aβ1-42, together with the Tau protein are two principal hallmarks of Alzheimer’s disease (AD) that are related to disease genesis and progression. Aβ1-42 possesses a higher aggregation propensity, and it is able to form fibrils via nucleated fibril formation. To date, there are compounds available that prevent Aβ1-42 aggregation, but none have been successful in clinical trials, possibly because the Aβ1-42 structure and aggregation mechanisms are not thoroughly understood. New molecules have been designed, employing knowledge of the Aβ1-42 structure and are based on preventing or breaking the ionic interactions that have been proposed for formation of the Aβ1-42 fibril U-shaped structure. Recently, a new Aβ1-42 fibril S-shaped structure was reported that, together with its aggregation and catalytic properties, could be helpful in the design of new inhibitor molecules. Therefore, in silico and in vitro methods have been employed to analyze the Aβ1-42 fibril S-shaped structure and its aggregation to obtain more accurate Aβ1-42 oligomerization data for the design and evaluation of new molecules that can prevent the fibrillation process.

## 1. Introduction

There are several proteins that can form water-insoluble aggregates in different cell lines, which have been generally named amyloids due to the similarity of their features with those reported by Sipe and Cohen, 2000 [1]. All amyloid proteins share a certain similarity in their amino acid sequences, and also in their secondary structure, in which β-folded structures predominate. These proteins have been associated with more than 100 diseases, including Alzheimer′s disease (AD), which is one of the most important neuropathologies [2].

Among the different pathophysiology theories of AD, the amyloid beta (Aβ) and hyperphosphorylated tau protein hypothesis is prominent due to direct evidence of neurotoxicity [3,4]. The original Aβ hypothesis states that accumulation of Aβ in the brain is the primary event that drives AD pathogenesis [5,6]. But, it has been shown that others physiopathology deleterious process occur simultaneously, perpetuating or increasing the damage caused by Aβ [7,8].

The most prevalent forms of Aβ are peptides with 40 (Aβ1-40) and 42 (Aβ1-42) amino acid residues, with the latter being the most toxic form. The Aβ1-42 peptide is formed from the amyloid precursor protein (APP) that is processed and cleaved by β- and γ-secretase [9]. Aβ1-42 is able to undergo several conformational changes after being delivered from the membrane; environmental conditions (pH, salts, and proteins) then enable Aβ1-42 to adopt a β-sheet structure and form neurotoxic oligomers and fibrils. The development of AD is associated with Aβ1-42 aggregation due to small oligomers that are formed during the early stages of aggregation, are neurotoxic and are involved in the process of neurodegeneration. However, Aβ has several physiological roles. One of its most important roles is to depress excess synaptic activity [10]; other functions that have been proposed are: has a role as link between kinesin and synaptic vesicles, maybe acting as adhesion protein, also participated in metal ion homeostasis and promotion of neurite growth [11,12], additionally, it could be an important component in the protection of the central nervous system against infections [13,14]. Not all of the Aβ physiological activities have been elucidated, but it is evident that it performs an important function in neuronal survival. However, the imbalance of Aβ1-42 production and degradation transforms it into a neurotoxin [15].

The monomers of Aβ1-42 self-assemble into a quaternary structure, adopting several structures. When fibril is formed this could be a β strand-turn β-strand structure. To acquire this conformation, there are several residue side chain interactions that favor the formation of parallel folded β structures with a hydrophobic core. These structural properties were also obtained for Aβ1-40 fibril. It has been proposed that the β-cross structure of Aβ1-42 is double-layered [16]. The Aβ1-42 oligomers and fibrils vary in their conformation depending on the initial process that originates them; therefore, Aβ1-42 oligomers and fibrils can show different shapes, although they arise from the same polypeptide amino acid sequence [17].

Therefore, the folding of Aβ1-42 in oligomers and fibrils, and the transition between these structures, is a complex process with origins in different polymorphs and consequently has several biological effects, some of which are correlated with that observed in brain samples of AD patients. Therefore, understanding the Aβ1-42 aggregation process is essential for the development of drugs that can prevent oligomer formation, or that could be used as early tracers (e.g., Pittsburgh Compound-B) that could detect the first fibrillation process in patients with a high risk of developing AD [18,19]. In this sense, in silico studies have clarified the folding and assembly of Aβ1-42 monomers to form oligomers, depicting how some molecules could interfere in Aβ1-42 folding, and aggregation. Correlating in silico studies with in vitro studies, such as atomic force microscopy (AFM), infrared spectroscopy, X-ray diffraction, transmission electron microscopy (TEM), and nuclear magnetic resonance in the solid-state (ssNMR), has contributed to the understanding of the kinetics of fibril formation. The properties of each of these techniques for the characterization of the Aβ1-42 fibrillation process are described in Table 1.

Experimental techniques, such as fluorescence, circular dichroism spectroscopy, and Fourier transform infrared spectroscopy, are widely used to monitor development of the Aβ1-42 β-sheet structure, but these methods do not provide atomic information on the aggregate Aβ1-42 tertiary or quaternary structure. Structural studies can be carried out using X-ray crystallography, TEM, and ssNMR, which give useful information on protein structure at the atomic level, such as backbone conformations, supramolecular organization, and inter-strand arrangements of the Aβ1-42 fibrils [32,44].

Recently, a new atomic model of the Aβ1-42 amyloid fibril based on ssNMR data was reported, where it displays triple parallel-β-sheet segments that are different from the reported structures of the Aβ1-40 fibrils. In addition, these ssNMR experiments suggested that the Ala42 carboxyl terminus, absent in Aβ1-40, forms a salt bridge with Lys28 to acquire the Aβ1-42 fibril S-shape. Furthermore, secondary nucleation processes catalyzed by the S-shape Aβ1-42 fibrils have been proposed, since the structure’s hydrophobic characteristics endow Aβ1-42 with catalytic properties. These facts are of great importance to the understanding of the structure and were elucidated by in silico and in vitro assays [45,46].

Therefore, in this review we will focus on the in vitro and in silico studies that have thus far established important structural and catalytic surface activity information of the Aβ1-42 structure based on its S-shape, such as ssNMR, EM, and electrophoresis, which allow the identification of the molecular weight of aggregates. We will also discuss the in silico studies, such as molecular dynamics (MD) simulations, which have given insights related to the S-shape of Aβ1-42. The physicochemical and biochemical techniques employed to characterize Aβ1-42 aggregation are highly important to the resulting structural characterization of the oligomers and fibrils of Aβ, and the structural characterization often depends on the information that is desired to be obtained.

### 1.1. Structural Properties of Aβ1-42 in Relation to Aβ1-40

**Figure 1:**
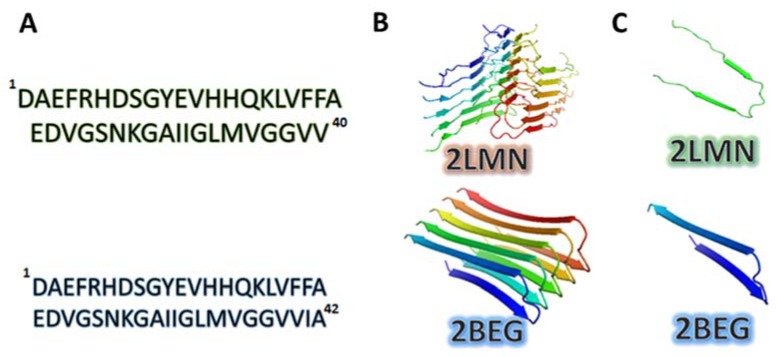
3D structure of Aβ1-40 and Aβ1-42. (**A**) Amino acid sequences for Aβ1-40 and Aβ1-42; (**B**) 3D structure of Aβ1-40 hexamer (PDB 2LMN) and Aβ1-42 pentamer (PDB 2BGE); (**C**) 3D structure of a monomer taken from each structure where the U-shape is observed.

During the hydrolysis of APP in the amyloidogenic pathway different long Aβ peptides are produced; however, Aβ1-40 and Aβ1-42 have been implicated in AD. Aβ1-42 is more toxic than Aβ1-40 [47,48]. Although these peptides only differ in two amino acid residues (Figure 1A), this is enough to induce different structural conformations despite being in the same environmental conditions.

The kinetics of Aβ1-42 fibril formation is via nucleated fibril formation [49,50]. This, includes three phases, in the first phase named the lag phase, the unstructured monomers form oligomers of several sizes with different structures, to date, some reports mention that these oligomers could be rich in β-sheets [51]. However, there are other works that mention that the dimers do not have high β-sheet content. At the end of the first phase, the oligomers form a nucleus, and after, in the elongation phase, occurs the addition of monomers to the original nucleus to make fibrils, this phase is faster and also produces fibrils of different morphology, depending on the experimental conditions [52,53]. During the fibril elongation process, the addition of a monomer with high β-sheet content is not maintained in the fibril, which suggests that the structure of oligomers or monomers are not maintained when binding to the fibril, due to several conformational changes that are involved [54].

It is known that during Aβ1-42 aggregation, each monomer is linked through hydrogen bonds, forming a protofibril of 25–30 Å, the accumulation of several protofibrils creates fibrils of 60–80 Å. The Aβ1-42 is the dominant Aβ species in the amyloid plaques of AD patients [55]. Although it has been proposed that the concentration ratio of Aβ1-42 to Aβ1-40 could increase during AD in some instances, this has not been found to be the case due to a lower ratio of Aβ1-42 to Aβ1-40 observed in plasma. This fact is an indicator of AD because it suggests the depletion of water-soluble Aβ1-42 due to its aggregation, producing the Aβ1-42 plaques in AD patients [56].

Aβ1-42 typically displays a higher propensity to form amyloid fibrils in vitro, producing different polymorphs that represent rearrangements of the molecular structure. Each of these could produce different biological activities, which highlights the importance of Aβ amyloid fibril characterization [57,58]. However, most high-resolution structural studies have been performed with Aβ1-40 amyloid fibrils and have identified the important structural Aβ1-40 characteristics [59,60], including the following:

(a) A structure with a U-shape (Figure 1B,C);
(b) A U-shape formed by two parallel β-sheets connected by a short, curved loop region (between residues Asp23 and Gly29, Figure 1C);
(c) A U-shape stabilized by the formation of a salt-bridge between the side chains of Asp23 and Lys28.

Similar structural properties were also reported for the Aβ1-42 fibril such as those reported for the PDB 2BGE structure:

(a) Protofibril or a cross-β-subunit, Figure 1B);
(b) Structure formed by two β-strands: β1 (residues 18–26) and β2 (residues 31–42);
(c) β1 and β2 connected by a hairpin loop (residues 27–30, Figure 1C), which allows formation of the Aβ1-42 U-shape structure (Figure 1C).

However, recent studies that have employed ssNMR have demonstrated that the Aβ1-42 fibril could have other interesting structural details and adopt an S-shape instead of a U-shape.

Nevertheless, all structural characteristics from the Aβ1-42 fibril have been supported in in vitro studies employing synthetic Aβ1-42. Because, despite of the many efforts made to observe and understand how the Aβ1-42 aggregation process is carried out in the human brain, some of them have not been successful. Currently, in vivo imaging studies and ex vivo histopathological studies only allow us to determine the presence of Aβ with an approximation of its state of aggregation [61,62]; but it is not possible to determine the atomic structure of the Aβ, nor to determine the aggregation stages in vivo, because the experimental conditions of extraction and purification of proteins could modify the quaternary structure [63,64]; therefore, it has not been possible to elucidate a quaternary structure for oligomers or fibrils in vivo in the brains of AD patients. Furthermore, it has not been confirmed whether the aggregation of Aβ in vitro is similar to those that occur in animal models and in patients. That is the reason that explains why in vitro studies are of great relevance, since when administering synthetic Aβ in vitro in neuronal and astrocyte cultures, as well as in animal models, it reproduces biochemical alterations similar to those determined in brain samples of patients with AD [65].

### 1.2. Aβ1-42 Fibril Preparation

The in vitro preparation of synthetic Aβ1-42 fibrils is difficult under laboratory conditions, considering the natural environmental conditions that lead to their formation [66]. The Aβ1-42 S-shape structure was discovered and published by Xiao et al., who developed a protocol to prepare the Aβ1-42 fibrils [45]. In this case, the samples were prepared by incubating Aβ1-42 solution for 24 h with the addition of 5% (*w*/*w*) of seeded amyloid fibrils. The reproducible preparation of Aβ1-42 fibril samples was made possible by careful optimization of the purification protocol, sample concentration, and incubation times. In this study, it was possible to obtain the seeded fibrils by repeating the seeding protocol three successive times after an initial incubation without a seed [45].

In addition, it has been reported that the fibril structure could be modulated by changes in buffer salts and concentrations. The substitution of 100 mM KCl for NaCl produces most of the fibrils grown in NaCl, and these have spiral structures. However, the number of hybrid fibrils increased in the presence of KCl, which could be due to an increase in the switching frequency (spiral to hybrid) or in the speed of fibril growth without a change in the switching frequency. The fibrils grow depending on the initial fibril nucleus structure, but switching from a straight to spiral mode is also possible because this switching phenomenon is affected by the buffer salt composition. This indicates that polymorphisms in fibril structure can occur after fibril nucleation, and it is affected by relatively modest changes in environmental conditions [32]. Therefore, the obtained structure of Aβ1-42 could be influenced by the experimental conditions.

In a micelle or in an apolar solvent environment the monomers of Aβ1-42 are largely disordered, but adopt an α-helical structure in the central hydrophobic core region and in the C-terminal region [67]. In water (pH 7.3) at room temperature and in the solvent concentration range between 5 and 10 mM, the Aβ1-42 peptide appears to adopt more β-sheet structure (79% β-sheet) [68]. Under more physiological conditions that are associated with AD, little α-helical structure appears to be present [69]. Therefore, the β content of the monomer is crucial for further aggregation but is sensitive to environmental conditions. Therefore, the dissolvent that is employed to generate the Aβ1-42 fibrils is important and can produce different aggregates, and several polymorph fibrils can arise due to the influence of salt content, polarity, and pH on the fibrillation process. Furthermore, Roche J et al. reported that the solvent composition influences the Aβ1-42 secondary nucleation and fibril growth because the NMR signal disappeared to a greater extent in D2O than in H2O due to the intermolecular interactions between the hydrophobic regions of the peptide [70].

In this way, the use of Aβ1-42 in the development of AD models could produce different results due to the preparation of Aβ1-42 fibrils and the lack of characterization of the administered Aβ1-42 aggregates. Therefore, an approximation of physiological conditions is needed to consistently obtain Aβ1-42 fibrils from synthetic Aβ1-42. Well-characterized Aβ1-42 species, such as oligomers, protofibrils, and fibrils, should be administered to induce a significant decrease in memory and an impairment of synaptic plasticity, a decreased number of viable neurons, increased tau levels, and a decreased number of dendritic spines; at this point researchers would have a well-established rat AD model [71]. Thus, experimental conditions in the Aβ1-42 oligomers and fibril preparation are important, not only to show a well-defined characterization but also to induce similar effects when the peptide is administered to animals. Recently, it was reported that Aβ1-42 not only acquires a U-shape, as do Aβ1-40 fibrils, it is also able to form S-shape fibrils. Despite this knowledge, to date, there has been no report on the administration of S-shape fibrils to the rat hippocampus nor have there been any reports on the differences between the well-characterized U-shape and S-shape fibrils in vivo.

### 1.3. Molecular Structure of S-Shape Aβ1-42 by Solid-State Nuclear Magnetic Resonance (ssNMR)

The molecular structure of Aβ1-40 has been analyzed more often in relation to Aβ1-42, whose structural details are poorly defined [59,72]. Aβ1-42 fibrils typically show structural and morphological heterogeneity due to its high propensity for misfolding [73].

**Figure 2:**
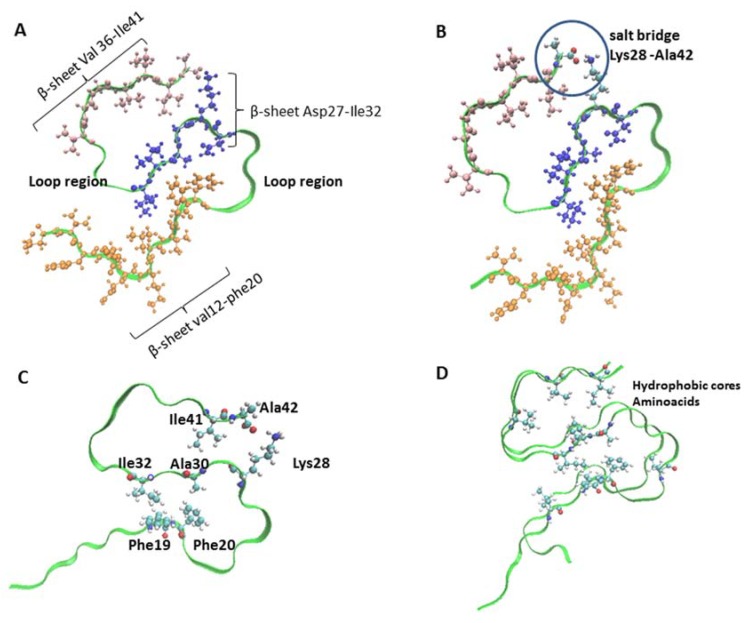
Triple-stranded β-sheet indicative of the S-shape fibril structure of Aβ1-42. (**A**) Val12–Phe20, Asn27–Ile32, and Val36–Ile41 are connected by two loop regions at Ala21–Ser26 and Gly33–Met35. PDB 2MXU; (**B**) salt bridge between Lys28 and Ala42. PDB 2MXU; (**C**) PDB ID: 5KK3; (**D**) Hydrophobic core amino acids in PDB 5KK3 dimer.

Recently, an ssNMR study of Aβ1-42 fibrils was reported, and additional important structural details were observed. The study employed a single conformer of Aβ1-42 fibril that was obtained by the seeding process and was verified by observing chemical shifts (the precise values of NMR frequencies), which showed a strong dependence on the local molecular conformation. A single set of chemical shifts for each residue implied that the Aβ1-42 in the fibril had only one conformer, and the fibrils showed three stretches of in-register parallel β-sheet regions that were formed by Val12–Phe20, Asn27–Ile32, and Val36–Ile41 and were connected by two loop regions at Ala21–Ser26 and Gly33–Met35 (Figure 2A). Additionally, inter-strand distance measurement for the Aβ fibril samples that were selectively labeled at ^13^CO of Ala30 and Leu34 indicated CO–CO distances of 5.0 Å ± 0.1 Å at both residues [45]. This Aβ1-42 structure differs from those reported for Aβ1-42 in the U-shape.

Furthermore, the intramolecular distance of 4.5 Å between the CO of the terminal carboxyl group of Ala42 and the N of the side chain of Lys28 implies a salt bridge formation between these groups (Figure 2B). This was also confirmed by frequency-selective rotational-echo double resonance (FS-REDOR) NMR, which is a high-resolution solid-state NMR technique employed to measure the dipolar coupling between a heteronuclear spin pair, confirming that a salt bridge exists between Lys28 and Ala42 (Figure 2B,C) [46]. This fact is the most interesting to date, because the stabilization by this salt bridge between Lys28 and Ala42 explains why the unique S-shaped triple-stranded β-sheet is only observed for Aβ1-42 fibrils, as Ala42 does not exist in Aβ1-40, indicating that the S-shape is not stable in Aβ1-40.

Additionally, the S-shape structure of the Aβ1-42 fibril has been identified in different studies, combining data from ssNMR spectroscopy and using mass-per-length (MPL) measurements from EM. The 3D structure is composed of two Aβ1-42 molecules per fibril layer, in which residues 15–42 are able to form a double-horseshoe-like cross-β-sheet, with hydrophobic side chains in the interior (PDB 2NAO) [74]. In addition, other studies employing NMR have shown that the fibril core consists of a dimer of Aβ1-42 molecules, each containing four β-strands in an S-shape and arranged in a manner that generates two hydrophobic cores that are capped at the end of the chain by a salt bridge between Lys28 and Ala42 (PDB 5KK3). The outer surface of the monomers presents hydrophilic side chains to the solvent. The interface between each one of the dimer shows clear contacts between Met35 of one monomer and Leu17 and Gln15 of the other monomer. The intermolecular interactions demonstrate that the amyloid fibrils are parallel (Figure 2D) [44]. Furthermore, Ile41–Gly29, Ile41–Lys28, Phe19–Ile32, Phe20–Val24, and Phe19–Ala30 form intramolecular interactions that form two hydrophobic pockets and, together with the salt bridge between the Ala42 and the Lys28, maintain the monomeric Aβ1-42 in an S-shape (Figure 2C) [44].

The fibril subunit illustrated in Figure 2D is a dimer formed from two S-shaped monomers that has most of the hydrophobic residues hidden within the fibril core. Two distinct hydrophobic cores are formed, one containing residues Ile31, Val36, Val39, and Ile41, and the other containing Leu17, Phe19, Phe20, Val24, Ala30, and Ile32. The latter is bridged by Met35 and Leu34 of both monomers to form one continuous hydrophobic cavity across the monomer interface. Some of the important interactions that determine the folding of the monomer and dimer structure are shown in Table 2.

As mentioned previously, the hydrophobic interactions are important during fibril formation, but these hydrophobic regions also have an important role during the secondary nucleation process. Additionally, the Aβ1-42 oligomers can be formed on the catalytic surface of the fibrils and increase the toxicity of the oligomers. It has been suggested that the interaction between the Aβ1-42 monomer and the fibril surface is of a hydrophobic and electrostatic nature due to the charged N-terminus of the Aβ1-42 monomer binding to the surface of the fibril between residues Ala30–Ile32.

It has been reported that during the initial step of the secondary nucleation process, the complete unfolding of the monomer in the C-terminal residues occurs in the fibril region formed by Ala30–Leu34 [46]. After the binding of the monomer on the fibril surface, the electrostatic interactions decrease between the monomer and fibril, supporting the idea that electrostatic interactions are important during the first step of secondary nucleation, whereas hydrophobic interactions drive the subsequent steps. Experimental studies of Aβ1-42 fibrillation reveal that the protofilaments are stable for several hours as a result of monomer Aβ1-42 aggregation onto the surface of the existing protofilaments [75]. Therefore, blocking the hydrophobic regions of the fibril surface could be an effective strategy to control the formation of neurotoxic Aβ1-42 oligomers [50].

The most recently published data regarding the Aβ1-42 fibril structure reveals a different structure compared to Aβ1-40 fibril. However, the presence of a salt bridge between the Lys28 and a carboxylic acid group is conserved, although the carboxylic position in the C-terminal region of Aβ1-42 can be stabilized by the S-shape. Electrostatic and hydrophobic interactions are important to stabilize the Aβ1-42 structure and to catalyze the secondary nucleation process. Although it has been reported that S-shape fibrils can catalyze the secondary nucleation process, more information is needed.

### 1.4. Molecular Structure of Aβ1-42 by Electron Microscopy (EM)

Electron microscopy (EM) has been applied successfully to determine the structures of many types of protein filaments. Scanning transmission electron microscopy (STEM) has proven to be a powerful tool in the investigation of the assembly mechanisms and structural properties of amyloids [76]. A high-resolution scanning TEM (STEM) image of Aβ1-42 fibrils shows twisted single strands with a diameter between 6 ± 1 nm and 13 ± 1 nm and exhibits thinner filaments between approximately 4.5 and 6.0 nm. The S-shape was reported in this study, but the relation MPL measurements were not reported [45]. However, in another study of the Aβ1-42 fibril, an average MPL value of 23.5 ± 0.1 kDa/nm was reported. Using the theoretical molecular mass of an Aβ1-42 molecule (4514 Da), the recorded MPL value translates to 2.44 Aβ1-42 molecules for each 4.7 Å repeat of the fibril [72]. In addition, these data are in accordance with those obtained by Colvin et al., where the MPL curves corresponded to 2.26 and 4.47 molecules/fibril subunit. Thus, the STEM measurements are consistent with the dimeric structure and a tetrameric structure with 2-fold symmetry. The latter corresponds to a fibril in which two filaments wind around each other, as seen by cryo-EM [44].

An interesting study of high-speed atomic force microscopy (HS-AFM) was able to demonstrate the initial fibril nucleation and subsequent fibril elongation. Distinct growth modes for Aβ1-42 fibrils were reported—one producing straight fibrils of ~5 nm in height and another producing spiral fibrils with ∼100-nm periodicity that varied in height between 5 and 10 nm. A hybrid structure was also found in which the spiral and straight structures coexisted [32].

Although EM has been widely employed to determine a morphological characterization of the Aβ1-42 oligomerization process, several studies have also used atomic force microscopy (AFM) in tapping mode to determine the morphology and size of the Aβ1-42 aggregates [77]. Different Aβ1-42 oligomers have been characterized prior to being administered to rats; however, the observed effects depend on the Aβ1-42 aggregate morphology which is formed at different times and may not depend on the peptide concentration [50].

### 1.5. Biochemical Techniques to Determinate AB1-42 Aggregation

Although electrophoresis, Western blot, and spot blot do not allow for structural determination, these experimental techniques are important to determine Aβ1-42 oligomerization. Aβ1-42 can be separated in a polyacrylamide gel based on its molecular weight because the proteins are amphoteric molecules that have both positive and negative charges. Sodium dodecyl sulfate polyacrylamide gel electrophoresis (SDS-PAGE) has played a central role in biochemical fractionation procedures. Its role in assessing the purity of antigen or immunoglobulin preparations, together with defining the molecular weights of target antigens by immunoprecipitation or immunoblotting (western blotting or spot blot) techniques, has given it an important role in immunochemistry [78].

The gel electrophoretic techniques were first used to identify the likely pathogenic role of Aβ1-42 deposits, and subsequently a gel system capable of resolving Aβ peptide variants was established for analyzing Aβ peptides from natural sources. Additionally, SDS-PAGE was capable of detecting the dimeric isoforms of Aβ [79].

Due to the tendency of Aβ to self-aggregate and form oligomers, it is well-known that when using SDS-PAGE, some of the smaller Aβ peptides migrate slower than their larger homologs [79,80,81]. The standard electrophoretic techniques in general are limited to larger proteins, because small peptides (~10 kDa) do not have a strong relationship between their mass and electrophoretic mobility [82,83].

It has been observed that with or without the use of SDS or 8 M urea the Aβ peptide becomes 100% random coil and remains monomeric, and the aggregation decrease. Nevertheless, it has been observed that the peptide and its variants do not obey the standard mass/mobility relationship in this medium [84].

Another SDS-PAGE type is the tris-glycine gel; the mobility of small peptides may be anomalous in this system [84]. A new approach is based on the separation of the protein and the Aβ peptides by preparative SDS-PAGE. Preparative gel electrophoresis was established by Lewis, Racusen, and Jovin in the early 1960s [85,86]. The approach was based on a modification of disc electrophoresis to isolate pituitary hormones [87] or hemoglobin [88].

The use of SDS-PAGE does not allow for the real-time monitoring of the aggregation kinetics, and its resolution remains limited, particularly for the high molecular weight species [89,90]. Another interesting separation technique is capillary electrophoresis (CE) with UV detection. This method allows for monitoring soluble populations of Aβ1-42. Fluorescence correlation spectroscopy, dynamic light scattering, filtration, and SDS-PAGE have been used to estimate the size of aggregates or oligomers and correlate them with neuroviability [89,91].

Brinet et al. developed an innovative, nondenaturing method of the time-dependent Aβ1-42 oligomerization pattern based on electrospray differential mobility analysis (ES-DMA), with a goal of providing direct, real-time characterization of the early, metastable, and neurotoxic species [92]. They observed that at the beginning of the in vitro oligomerization process, the size distribution of Aβ1-42 is characterized by two populations, corresponding to the monomer and small oligomers (small diameters), and after a few hours the larger species are observed (approximately 10 nm) [92].

It is well-known that the characterization of Aβ oligomers is challenging, since it covers many different aggregation states that form in a time-dependent manner. In this sense, electrospray ionization mass spectrometry (ESI-IM-MS) is a technique that provides qualitative (structural) and quantitative (molecular mass or concentration) information for oligomer characterization, where it is possible analyze the molecules after their conversion to ions [93]. According Pujol et al., ESI-IM-MS analysis revealed that Aβ1-40 and Aβ1-42 predominantly oligomerize through dimers and trimers [45].

Monomeric and dimeric Aβ1-42 species in the Alzheimer’s disease brain homogenates have been observed by Western blot [94]. It is important that the fibrils from synthetic Aβ be recognized by specific antibodies that bind to senile plaques from AD patient brain samples, such as mOC1, mOC3, mOC16, mOC23, and mOC24, and not be recognized by other antibodies that do not bind to plaques, such as mOC9, mOC1 5, mOC22, mOC29, and mOC31, which are not able to detect intracellular deposits and senile plaques in human Alzheimer’s patients [44].

### 1.6. In Silico Studies Employing the S-Shape Aβ1-42 Structure

The most recent S-shape Aβ1-42 fibril structure has been used in in silico studies to analyze its catalytic surface properties, its aggregation mechanism, and its structural stability in relation to a U-shape. PDB 5KK3 is one of the most employed S-shape Aβ1-42 fibril structures. It consists of two filaments and has been used to analyze whether an S-shaped filament facilitates the formation of new S-shaped oligomers by simulating a peptide monomer/dimer placed in the proximity of the S-shaped filament. The in silico studies have shown that a monomer does not assume the S-shape conformation even when in proximity to a long filament of S-shape Aβ1-42 peptides. Remarkably, a dimer of Aβ1-42 peptides showed stability and retained its S-shape conformation. This in silico study showed the stability of an Aβ1-42 dimer that interacted with a long filament, which is consistent with the in vitro experimental study where fibrils facilitated the nucleation of Aβ1-42 peptides, simultaneously using two monomers in the initial step of oligomer formation [95]. However, Zahng et al. reported that the probability that Aβ1-42 dimers exist in a U and S shape is very low [96].

Aβ1-42 has high and fast kinetic propensity to form fibrillation due its lower barrier of nucleation, favoring the addition of Aβ1-42 monomers. The elongation process of Aβ1-42 monomer addition to form the fibril is kinetically favorable. Once the nucleus is formed, the addition of more monomers is thermodynamically favorable until the formation of a macroscopic fiber [68]. This process resembles that described by Elser et. al. who show that the grown of the fibril occurred by the addition of a monomer to the fibril, being the first step the binding of the monomer to the fibril (dock process) which is faster than second step which requires several conformational changes to allow the binding of the monomer to the growing fibril (lock process) [97]. Then, the dock-lock process is involved during the elongation fibril process, however, when the lock process is incomplete the delivery of monomer could conduce the new formation of oligomers.

The stability of S-shape Aβ1-42 has been analyzed by dynamics studies using replica-exchange molecular dynamics (REMD) simulations to compare the conformations of Aβ1-42 fibrils in the U-shape and S-shape. The computational results showed that the S-shape model is more stable than other Aβ1-42 shapes due to the interactions involving the C-terminal residues. The U-shaped model suffers significant distortions, resulting in a more disordered assembly. This could be explained by the intra-chain salt bridge linking the side chain of Lys28 with Ala42. The S-shape model appears to be subjected to a partial distortion only in the N-terminal region (Leu17-Asp23 region). Furthermore, the hydrophobic contacts in Aβ1-42 generated by the C-terminal residues Ile41 and Ala42 favored its stability in relation to Aβ1-40. Therefore, the Aβ1-42 S-shape fibril is the most stable structure due to inter-chain hydrophobic contacts and H-bonds involving the C-terminal residues Ile41 and Ala42.

**Figure 3:**
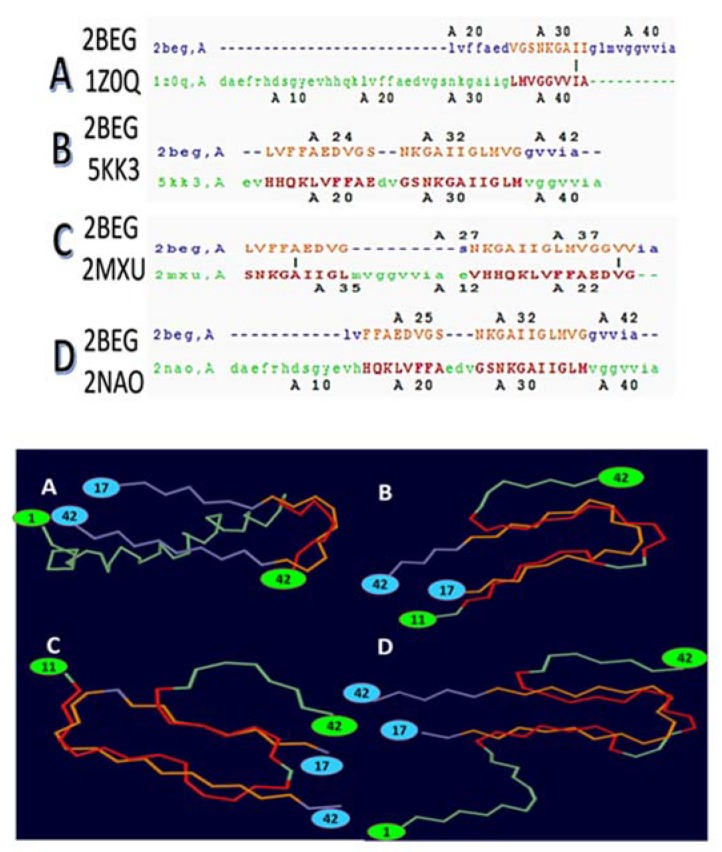
Alignment of the 2BEG structure (chain A; 17–42) with different structures. All the alignments show the 2BEG chain A in blue. (**A**) 2BEG chain A with 1Z0Q chain A; (**B**) 2BEG chain A with 5KK3 chain A (11–42); (**C**) 2BEG chain A with 2MXU chain A (11–42); (**D**) alignment of 2BEG chain A with 2NAO chain A (1–42).

Notably, the presence of N-terminal residues contributes to the S-shape stabilization due to the Glu11-Lys16 (PDB 2MXU, 5KK3) domain, which are thought to strengthen and further stabilize the inter-chain hydrogen bonds in comparison to the protein region Leu17-Val24 in PDB 2BEG (Figure 3). Thus, the S-shape 11-42 model shows a higher intrinsic order with respect to the U-shape 17-42 model (PDB 2BEG).

Grasso et al. [98] concluded that the S-shape structure is more stable than the U-shape, which contradicts the literature where the U-shaped model has been classified as rather stable. However, the S-shape structure is more complete because it possesses more amino acid residues in the N-terminal (PDB: 5KK3, 2MXU, and 2NAO; Figure 3) [98,99]. Therefore, several differences have been found in relation to the inter-sheet side chain contacts, hydrophobic contacts among the strands, and salt bridges in stabilizing the U- and S-shape protein aggregates [100].

The assembly of Aβ1-42 peptides can determine polymorphisms during oligomerization and fibrillization, but the mechanism of this effect remains unknown. Cheon et al. carried out a study starting with separate random monomers and using discontinuous MD simulations [101]. The trimer and tetramer stimulations drove the formation of nonfibrillar oligomers, unlike the pentamers and hexamers, both of which caused U-shape fibrillary structures. This is why pentamers and hexamers are called paranuclei. Furthermore, when the fibrillar oligomers were exceeded by the hexamers, it provoked substantial polymorphisms, in which hybrid structures were readily formed. Six of the hybrids were selected: one that was highly in-register and called “U-shape” (U), three S-shape (S1, S2, S3), and two other fibrillar structures (D1 and D2). The structures were compared with the well-ordered fibrillar PDB structures (2BEG and 2MXU) of the Aβ1-42 peptide in the U-shape and S-shape, respectively.

Interestingly, the S-shape structure (called S2) had a high similarity with 2MXU, which is a recently suggested Aβ1-42 fibril structure. Hence, the simulation successfully produced the S-shape structure. The U-shape structures, which have the lowest energy, can be easily formed, with significantly less trapping into other fibrillar structures. Among the 45 total U-shape fibrillar structures for both number chain (NC) = 5 and 6, 34 U-shape structures were converted from partial or full S-shape structures, which means that a small S-shape oligomer is not stable on its own. In the simulations, the pentamers or hexamers (paranuclei) are potent intermediates that are converted into fibrillar nuclei for further fibril growth [101].

Xi Wenhui et al. has reported that S-shape Aβ1-42 can assemble into ring-like structures that are stable during molecular dynamics simulations [102]. This model is stabilized by inter-chain salt bridges between residues Lys16 and either Glu22 or Asp23, which were identified by specific NMR signals. However, the assembled ring-like packing is energetically less favorable than that stabilized by contacts between residues Gly15 and Met35, which have been observed in the experimentally resolved Aβ42 fibrils with two-fold symmetry. Additionally, this model would have different responses to salt concentration or pH changes [102].

### 1.7. Structures Employed to Design Drugs 

The Aβ1-42 S-shape structure provides a tool for investigating molecules in vitro and in vivo that may have a destabilizing effect on this structure, and it can also be used to design new destabilizing molecules, taking into account the Aβ1-42 S-shape. Until now, only dihydrochalcone has been reported to destabilize the S-shape structure by binding to the protofibril cavity. A snapshot at *t* = 200 ns shows that dihydrochalcone molecules mostly bind to three sites: the protofibril cavity, the exterior of CHC (17Leu-Val-Phe-Phe-Ala-Glu-Asp23), and the C-terminal hydrophobic groove (31IleIleGlyLeuMet35). In addition, dihydrochalcone also binds to N-terminal residues 4Phe-Arg-His6 and 10Tyr12Val-His-His-Gln15. These results indicate that dihydrochalcone has similar binding sites and a similar destabilization effect on Aβ1-40 protofibrils as on Aβ1-42 [103].

## 2. Conclusions

Although the most reliable information from the Aβ1-42 structure should be that obtained from AD patients, little information is available about this because once the protein is taken from its natural environment, it can adopt altered structural conformations. The information provided by synthetic Aβ1-42 is of great help to more thoroughly understanding the Aβ1-42 structure and its oligomerization process. However, one of the principal problems of the structural studies lies in Aβ1-42 sample preparation, because under experimental conditions different aggregates are produced, which give rise to different results in animal models.

Recently, a single structure of Aβ1-42 was obtained after standardizing the Aβ1-42 seeding that showed a different structure from those obtained from Aβ1-40. The structure had an S-shape with a triple-parallel-β-sheet structure and a salt bridge between the Lys28 side chain and the Ala42 carboxyl terminus.

The newly discovered Aβ1-42 structure is clearly important for understanding the aggregation of Aβ1-42 and will be considered as a target for the rational design of effective compounds. Additionally, knowledge of the secondary nucleation process will help to design new molecules that have a high affinity for the surface of the fibril and can interfere with secondary nucleation. This new Aβ1-42 structural information may improve the possibilities for Alzheimer’s disease therapeutic treatments.

## Figures and Tables

**Table 1:** Spectroscopic and biochemical techniques used in the characterization of the Aβ1-42 structure and its fibrillation process.

Technique	Characteristics	References
Infrared spectroscopy (IR)	IR reveals the chemical bonds, peptide interactions, and β-sheet disposition of Aβ1-42.	[5,20,21,22,23]
X-ray diffraction	Shows details of the fibril structure, such as sheet direction and arrangements in amyloid crystals.	[6,24,25,26,27]
Microscopy transmission electron microscopy (TEM)	TEM allows determination of the ultrastructure organization throughout the electron–electron interaction in the Aβ1-42 structures at molecular level and atomic resolution.	[17,28,29,30,31]
Atomic force microscopy (AFM)	The resolution of this technique is less than 1 nm, enabling the structural details of Aβ1-42 aggregation to be revealed.	[32,33,34,35,36]
Fluorescence	Monitors Aβ1-42 aggregation kinetics in real-time and detects Aβ1-42 at any state in tissue samples using fluorochromes, such as Thioflavin T (ThT).	[37,38,39,40,41]
Electrophoresis	This technique could be used determine molecular weight and to purify Aβ1-42.	[5,20,40,42,43,44]

**Table 2:** Important intra- and intermolecular interactions during Aβ1-42 aggregation in the S-shape.

Intramolecular Monomer	
Amino acid residue interactions	Hydrophobic regions
Ile41-Gly29; Ile41-Lys28; Phe19-Ile32; Phe20-Val24; Lys28-Ala42	Ile31, Val36, Val39, Ile41, Leu17, Phe19, Phe20, Val24, Ala30, Ile32
Phe19-Ala30; Val24-Gly29; Ile31-Val36; Gly29-Asn27; Gly33-Val36; Gly29-Ile41	
**Intermolecular Dimer**	
Amino acid residue interactions	Hydrophobic regions
Met35-Leu17; Gln15-Leu31	Val18, Ala21, Val40, Val42

## Abbreviations

Aβ1-42	β-amyloid 1-42
APP	Amyloid precursor protein
AD	Alzheimer disease
Aβ	Amyloid beta
IR	Infrared spectroscopy
TEM	Transmision electron microscopy
AFM	Atomic force microscopy
ThT	Tioflavin T
SSNMR	Solid state nuclear magnetic resonance
FS-REDOR	Frequency selective rotational-echo, double resonance
MPL	Mass-per length
EM	Electron microscopy
STEM	Scanning transmission electron microscopy
HS-AFM	High-speed atomic force microscopy
SDS-PAGE	Sodium dodecyl sulfate polyacrylamide gel electrophoresis
CE	Capillary electrophoresis
ES-DMA	Electrospray differential mobility analysis
REMD	Replica Exchange Molecular Dynamics
